# A systematic review and meta-analysis to revise the Fenton growth chart for preterm infants

**DOI:** 10.1186/1471-2431-13-59

**Published:** 2013-04-20

**Authors:** Tanis R Fenton, Jae H Kim

**Affiliations:** 1 Alberta Children’s Hospital Research Institute, The University of Calgary, Calgary, AB, Canada; 2 Department of Community Health Sciences, The University of Calgary, 3280 Hospital Drive NW, Calgary, AB, Canada; 3 Division of Neonatology, UC San Diego Medical Center, 200 West Arbor Drive MPF 1140, San Diego, CA, USA

**Keywords:** Infant, Premature, Infant, very low birth weight, Preterm infant, Growth, Weight, Head circumference, Length, Percentile

## Abstract

**Background:**

The aim of this study was to revise the 2003 Fenton Preterm Growth Chart, specifically to: a) harmonize the preterm growth chart with the new World Health Organization (WHO) Growth Standard, b) smooth the data between the preterm and WHO estimates, informed by the Preterm Multicentre Growth (PreM Growth) study while maintaining data integrity from 22 to 36 and at 50 weeks, and to c) re-scale the chart x-axis to actual age (rather than completed weeks) to support growth monitoring.

**Methods:**

Systematic review, meta-analysis, and growth chart development. We systematically searched published and unpublished literature to find population-based preterm size at birth measurement (weight, length, and/or head circumference) references, from developed countries with: Corrected gestational ages through infant assessment and/or statistical correction; Data percentiles as low as 24 weeks gestational age or lower; Sample with greater than 500 infants less than 30 weeks. Growth curves for males and females were produced using cubic splines to 50 weeks post menstrual age. LMS parameters (skew, median, and standard deviation) were calculated.

**Results:**

Six large population-based surveys of size at preterm birth representing 3,986,456 births (34,639 births < 30 weeks) from countries Germany, United States, Italy, Australia, Scotland, and Canada were combined in meta-analyses. Smooth growth chart curves were developed, while ensuring close agreement with the data between 24 and 36 weeks and at 50 weeks.

**Conclusions:**

The revised sex-specific actual-age growth charts are based on the recommended growth goal for preterm infants, the fetus, followed by the term infant. These preterm growth charts, with the disjunction between these datasets smoothing informed by the international PreM Growth study, may support an improved transition of preterm infant growth monitoring to the WHO growth charts.

## Background

The expected growth of the fetus describes the fastest human growth, increasing weight over six-fold between 22 and 40 weeks. Preterm infants, who are born during this rapid growth phase, rely on health professionals to assess their growth and provide appropriate nutrition and medical care.

In 2006, the World Health Organization (WHO) published their multicentre growth reference study, which is considered superior [1] to previous growth surveys since the measured infants were selected from communities in which economics were not likely to limit growth, among culturally diverse non-smoking mothers who planned to breastfeed [2]. Weekly longitudinal measures of the infants were made by trained data collection teams during the first 2 years of this study [3]. These WHO growth charts, although recommended for preterm infants after term age [4], begin at term and so do not inform preterm infant growth assessments younger than this age.

Optimum growth of preterm infants is considered to be equivalent to intrauterine rates [5-7] since a superior growth standard has not been defined. Perhaps the best estimate of fetal growth may be obtained from large population-based studies, conducted in developed countries [8], where constraints on fetal growth may be less frequent.

A recent multicentre study by our group (the Preterm Multicentre Growth (PreM Growth) Study) revealed that although the pattern of preterm infant growth was generally consistent with intrauterine growth, the biggest deviation in weight gain velocity between the preterm infants and the fetus and infant was just before term, between 37 and 40 weeks (Fenton TR, Nasser R, Eliasziw M, Kim JH, Bilan D, Sauve R: Validating the weight gain of preterm infants between the reference growth curve of the fetus and the term infant, The Preterm Infant Multicentre Growth Study. Submitted BMC Ped 2012). Rather than demonstrating the slowing growth velocity of the term infant during the weeks just before term, the preterm infants had superior, close to linear, growth at this age. This finding has been observed by others as well [9-11]. Therefore, there is evidence to support a smooth transition on growth charts between late fetal and early infant ages.

Several previous growth charts based on size at birth presented their data as completed age, which affects the interpretation and use of a growth chart [12]. The use of completed weeks when plotting a growth chart requires all the measurements to be plotted on the whole week vertical axes. However, the use of completed weeks in a neonatal unit may not be intuitive, as nursery staff and parents think of infants as their exact age, and not age truncated to previous whole weeks. The advent of computers in health care, for clinical care and health recording, allow the use of the computer to plot growth charts, daily and with accuracy. It would make sense to support plotting daily measurements continuously by shifting the data collected as completed weeks to the midpoint of the next week to remove the truncation of the data collection as completed weeks.

The objectives of this study were to revise the 2003 Fenton Preterm Growth Chart, specifically to: a) use more recent data on size at birth based on an inclusion criteria, b) harmonize the preterm growth chart with the new WHO Growth Standard, c) to smooth the data between the preterm and WHO estimates while maintaining integrity with the data from 22 to 36 and at 50 weeks, d) to derive sex specific growth curves, and to e) re-scale the chart x-axis to actual age rather than completed weeks, to support growth monitoring.

## Methods

To revise the growth chart, thorough literature searches were performed to find published and unpublished population-based preterm size at birth (weight, length, and/or head circumference) references. The inclusion criteria, defined a priori, designed to minimize bias by restriction [13], were to locate population-based studies of preterm fetal growth, from developed countries with:

a) Corrected gestational ages through fetal ultrasound and/or infant assessment and/or statistical correction;

b) Data percentiles at 24 weeks gestational age or lower;

c) Sample of at least 25,000 babies, with more than 500 infants aged less than 30 weeks;

d) Separate data on females and males;

e) Data available numerically in published form or from authors,

f) Data collected within the past 25 years (1987 to 2012) to account for any secular trends.

### A. Data selection and combination

Major bibliographic databases were searched: MEDLINE (using PubMed) and CINHAL, by both authors back to year 1987 (given our 25 year limit), with no language restrictions, and foreign articles were translated. The following search terms as medical subject headings and textwords were used: (“Preterm infant” OR “Premature Birth”[Mesh]) OR (“Infant, Premature/classification”[Mesh] OR “Infant, Premature/growth and development”[Mesh] OR “Infant, Premature/statistics and numerical data”[Mesh] OR “Infant, very low birth weight”[Mesh]) AND (percentile OR *centile* OR weeks) AND (weight OR head circumference OR length). Grey literature sites including clinical trial websites and Google were searched in February 2012. Reference lists were reviewed for relevant studies.

All of the found data was reported as completed weeks except for the German Perinatal Statistics, which were reported as actual daily weights [14]. To combine the datasets, the German data was temporarily converted to completed weeks. A final step converted the meta-analyses to actual age.

### B. Combine the data to produce weighted intrauterine growth curves for each sex

**Figure 1:**
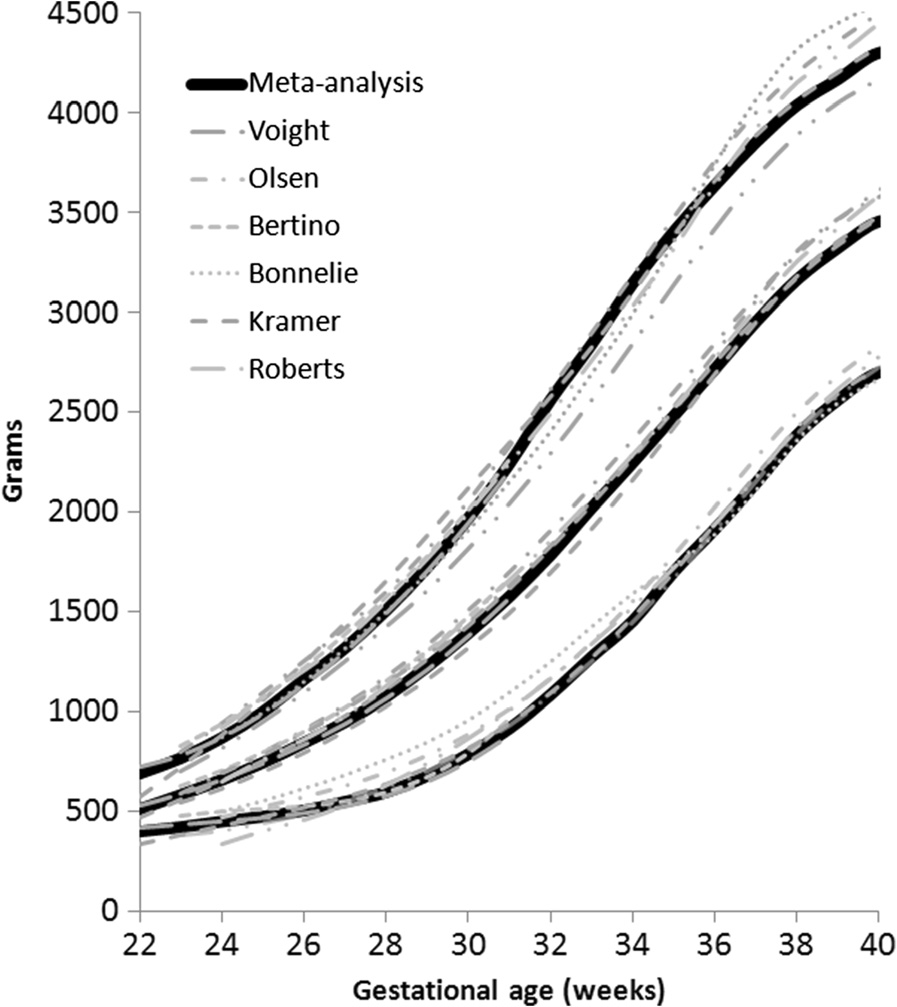
Boys birthweight centiles (3rd, 50th and 97th) from the six included studies, along with the boy’s meta-analysis curves (bold).

**Figure 2:**
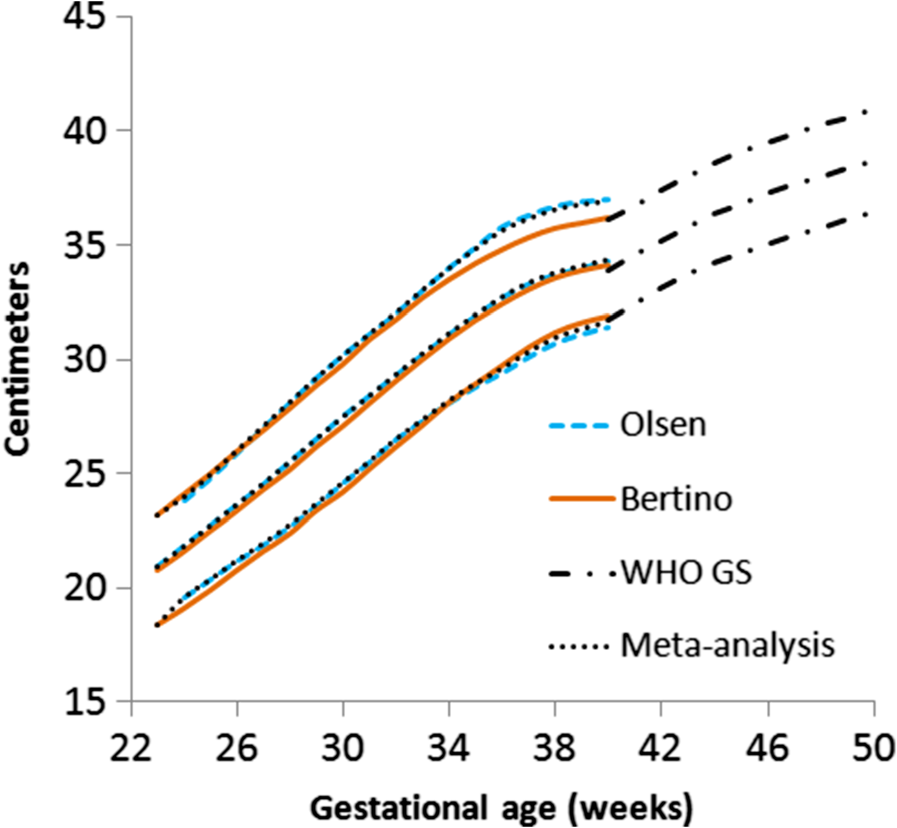
Girls head circumference centiles (3rd, 50th and 97th) centiles from the included studies, along with the girl’s meta-analysis curves (dotted), and after 40 weeks, the World Health Organization centiles (dashed).

**Figure 3:**
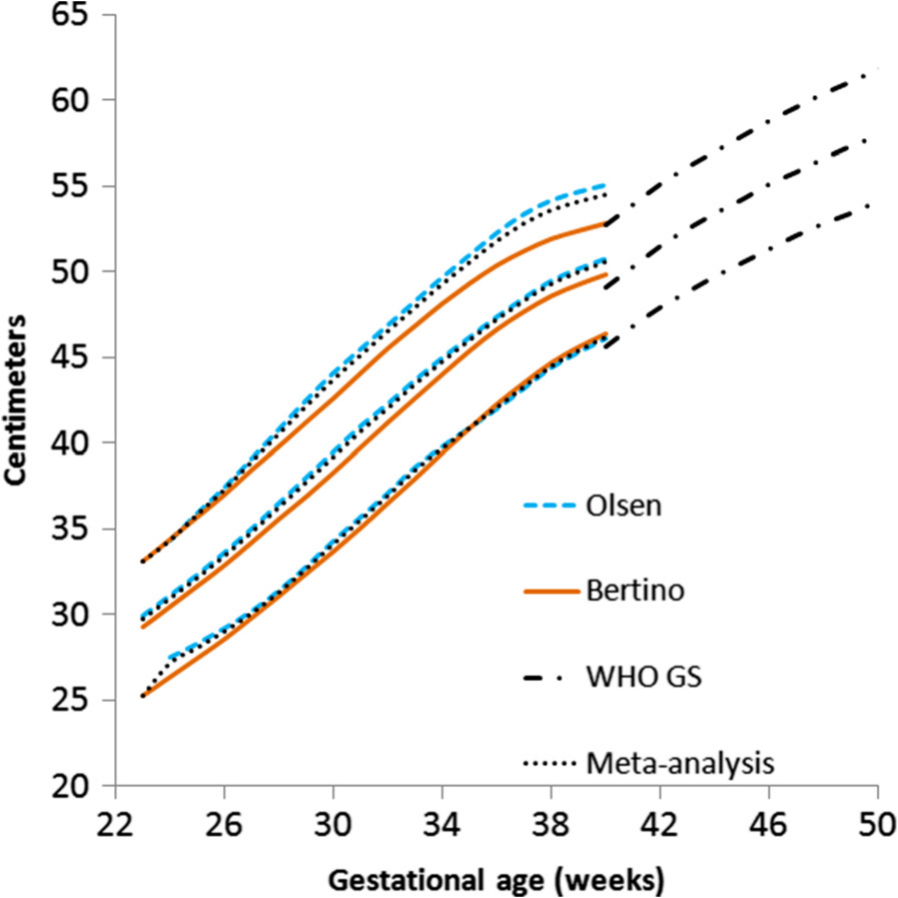
Girls length centiles (3rd, 50th and 97th) centiles from the included studies, along with the meta-analysis curves (dotted), and after 40 weeks, the World Health Organization centiles (dashed).

The located data (3rd, 10th, 50th, 90th, and 97th percentiles for weight, head circumference, and length) that met the inclusion criteria were extracted by copying and pasting into spreadsheets. The male and female percentile curves from each included data set for weight, head circumference and length were plotted together so they could be examined visually for heterogeneity (Figures 1, 2, and 3). The data for each gender were combined by using the weekly data for the percentiles: 3rd, 10th, 50th, 90th, and 97th, weighted by the sample sizes. The combined data was represented by relatively smooth curves.

### C. Develop growth monitoring curves

To develop the growth monitoring curves that joined the intrauterine meta-analysis data with the WHO Growth Standard (WHOGS) smoothly, the following cubic spline procedure was used to meet two objectives:

a) To maintain integrity with the meta-analysis curves from 22 to 36 weeks. Integrity of the fit was assumed to be agreement within 3% at each week.

b) To ensure fit of the data to the WHO values at 50 weeks, within 0.5%.

Procedure:

1) Cubic splines were used to interpolate smooth values between selected points (22, 25, 28, 32, 34, 36 and 50 weeks). Extra points were manually selected at 40, 43 and 46 weeks in order to produce acceptable fit through the underlying data. The PreM Growth study (Fenton TR, Nasser R, Eliasziw M, Kim JH, Bilan D, Sauve R: Validating the weight gain of preterm infants between the reference growth curve of the fetus and the term infant, The Preterm Infant Multicentre Growth Study. Submitted BMC Ped 2012) conducted to inform the transition between the preterm and WHO data, was used to inform this step. The Prem Growth Study found that preterm infants growth in weight followed approximately a straight line between 37 and 45 weeks, as others have also noted [9-11].

2) LMS values (measures of skew, the median, and the standard deviation) [15] were computed from the interpolated cubic splines at weekly intervals. Cole’s procedures [15] and an iterative least squares method were used to derive the LMS parameters (L = Box-Cox power, M = median, S = coefficient of variation) from the multicentre meta-analyses for weight, head circumference and length. The LMS splines were smoothed slightly while maintaining data integrity as noted above.

3) The final percentile curves were produced from the smoothed LMS values.

4) A grid similar to the 2003 growth chart was used, but the growth curves were re-scaled along the x-axis from completed weeks to allow clinicians to plot infant growth by actual age in weeks, and a slight modification (scaled to 60 centimeters instead of 65) was made to the y-axis.

### D. Compared the revised charts with the 2003 version

The revised growth charts were compared graphically with the original 2003 Fenton preterm growth chart. To make the differences in chart values more apparent, the 2003 chart data was also shifted to actual weeks for these comparison figures.

## Results

**Figure 4:**
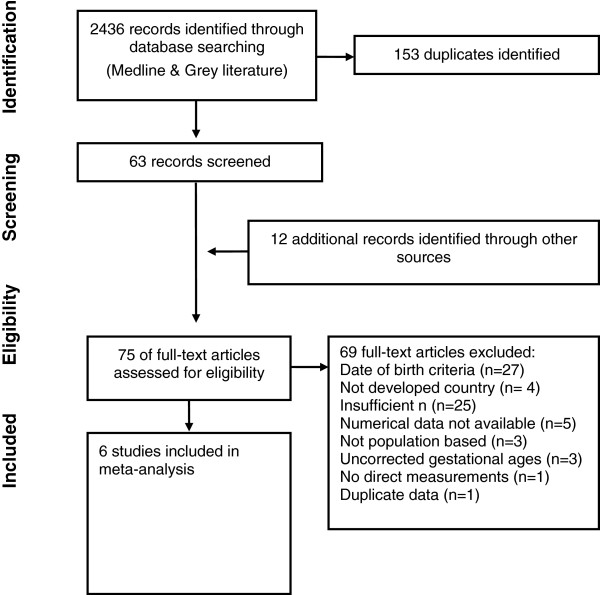
Flow diagram of studies identified, excluded and included in the systematic review.

Six large population based surveys [14,16-20] of size at preterm birth from countries Germany, United States, Italy, Australia, Scotland, and Canada were located that met the inclusion criteria (Table 1). The literature search identified 2436 papers, of which 2373 were discarded as being not relevant or duplicates based on the titles (Figure 4). Reviewing reference lists identified another 12 studies. Seventy-five studies were examined in detail, however 27 of these did not meet the date criteria. Among the 48 studies that met the date of birth criteria, some did not meet the other inclusion criteria for the following reasons: Did not meet the criterion for more than 25,000 babies [21-35], no low gestational age infants less than 25 weeks [31,36-41], insufficient number less than 30 weeks [34,42-45], no statistical correction for inaccurate gestational ages [46-48], numerical data not available [49-51], number of infants each week were not available [52], number of infants in the subgroups each week were not available [53], was not population based [54-56], no direct measurements [27], some of the data [57] was also in one of the larger included studies [17].

**Table 1:** Details of the data sources

	**Voight, 2010**	**Olsen, 2010**	**Kramer, 2001**	**Roberts, 1999**	**Bonellie, 2008**	**Bertino, 2010**	**WHO, 2006**
Data source	German Perinatal Survey	Pediatrix Medical Group hospitals	Canadian national file	Australian National Perinatal Statistics Unit	Scottish maternity data collection	Italian Neonatal Study	WHO multicentre growth reference study
Sample size	2,300,000	130,111	676,605	734,145	100,133	45,462	882
n < 30 weeks	14146	11377	3247	3193	2053	623	N/A
Lowest gestational age	22	23	22	20	24	23	term
Dates	1995 to 2000	1998 to 2006	1994 to 1996	1991 to 1994	1998 to 2003	2005 to 2007	1997-2003
Data	Weight	Weight, head, length	Weight	Weight	Weight	Weight, head, length	Weight, head, length
Exclusion criteria	None stated, included both live and stillborn	Multiple births, congenital anomalies, death before discharge, outlier measures (> 2 x interquartile range below the first and 3rd quartile).	Ontario province was excluded due to problems with data quality.	Omitted multiple and still births (births < 400 grams did not need to be recorded)	Multiple births, lethal anomalies, weights < 250 grams, and outlier measures (> 2 x interquartile range outside the first and 3rd quartile).	Multiple births, stillbirths, major congenital anomalies, and fetal hydrops	Maternal smoking, not breastfeeding, solids before 4 months. Screened for environmental or economic constraints.
Method to assess gestational age	Ultrasound assessment 8–14 weeks and Naegle’s rule.	Neonatologist assessment	“early ultrasound has increasingly been the basis for gestational age assessments in recent years”	Dates, prenatal, or postnatal assessment	Clinician assessment based on ultrasound, maternal dates, and clinical estimates	Ultrasound assessment first trimester	Not stated
Outliers/smoothing method	Cubic regression, LOESS smoothing, LMS parameter smoothing	LMS methods, with the skew set to one and further manual smoothing	Assumed a log normal distribution of birthweight at each gestational age and compared the probabilities of accurate versus misclassification of infant’s gestational age	Omitted outlier measures (> 2 x interquartile range below the first and 3rd quartile).	Cubic spline fitting	Generalized logistic functions	Omitted outliers > 3 SD, LMS parameter smoothing, skew set to one for weight, cubic spline fitting.

Included in the meta-analyses were almost four million (3,986,456) infants at birth (34,639 less than 30 weeks) from six studies for weight (Table 2), and 173,612 infants for head circumference, and 151,527 for length [16,18]. The World Health Organization data measurements were made longitudinally on 882 infants.

**Table 2:** Number of infants each week from each study

**Gestational age**	**Voight, 2010**		**Olsen, 2010**		**Bertino, 2010**		**Kramer, 2001**		**Roberts, 1999**		**Bonellie, 2008**	
	Females	Males	Females	Males	Females	Males	Females	Males	Females	Males	Females	Males
22	188	321	-	-	-	-	80	82	71	74	-	-
23	431	560	133	153	3	8	106	114	79	95	-	-
24	575	704	438	451	20	24	148	156	115	135	120	126
25	713	846	603	722	40	38	184	202	136	180	115	118
26	812	968	773	881	35	58	191	234	188	235	179	172
27	1073	1203	966	1030	52	61	188	254	231	284	174	177
28	1276	1536	1187	1281	79	63	287	330	287	361	246	239
29	1516	1838	1254	1505	70	72	299	392	325	397	245	265
30	1853	2212	1606	1992	107	114	390	467	440	571	317	313
31	2283	2956	2044	2460	126	140	461	584	548	743	136	148
32	*	*	3007	3677	165	183	795	997	877	1117	193	205
33	*	*	4186	5014	211	240	1055	1368	1200	1471	239	256
34	*	*	5936	7291	263	349	2018	2553	2086	2657	374	422
35	*	*	5082	6952	366	418	3391	4314	3418	4092	644	653
36	*	*	4690	7011	562	665	8203	9648	7320	8788	1048	1265
37	*	*	4372	6692	1291	1492	17308	19965	16105	18660	2006	2499
38	*	*	5755	8786	3524	3976	47516	51947	47809	51404	4630	6387
39	*	*	5978	8324	5295	5452	75068	77623	68846	72871	8699	10706
40	*	*	5529	7235	5672	5653	110738	112737	137570	141553	12644	14230

* Not reported.

**Figure 5:**
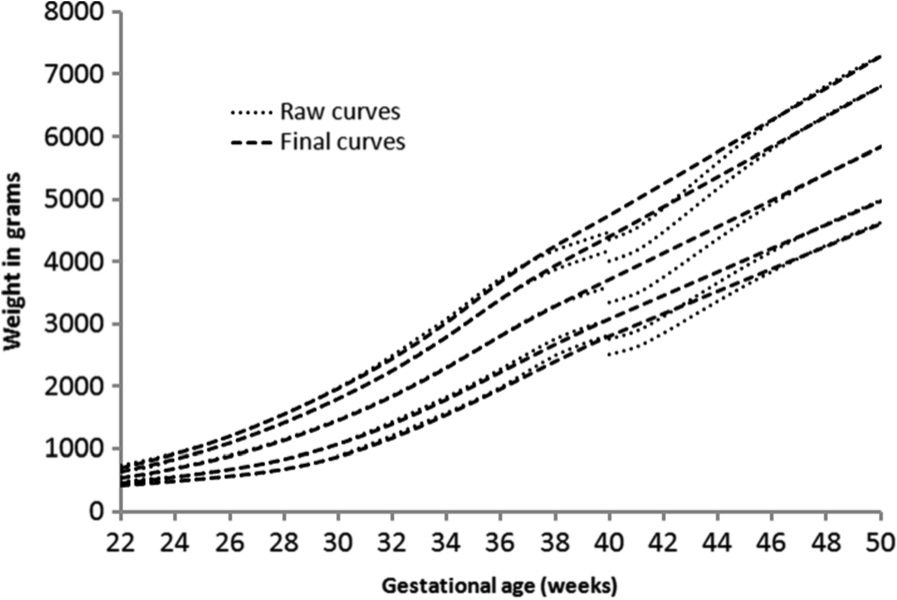
Boys meta-analysis weight curves (dotted) with the final smoothed growth chart curves (dashed).

**Figure 6:**
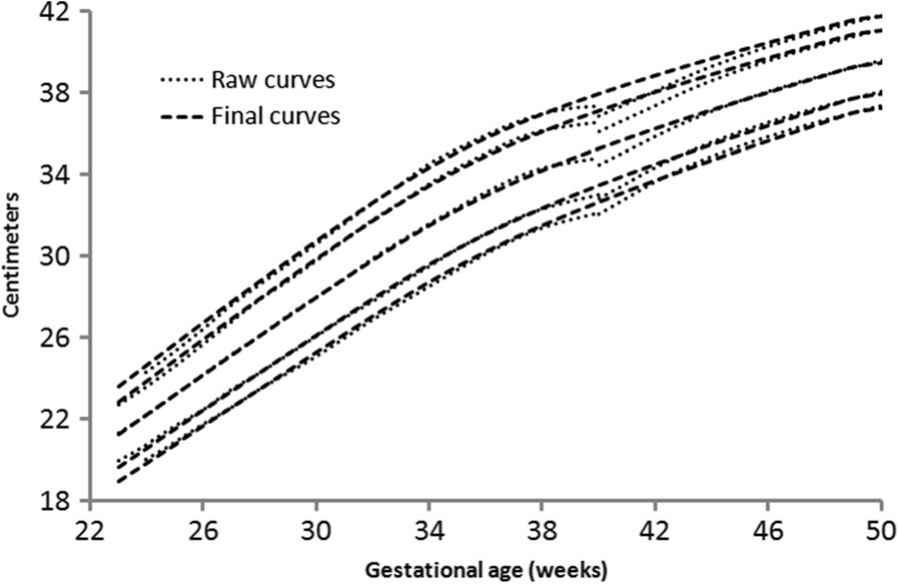
Boys meta-analysis head circumference curves (dotted) with the final smoothed growth chart curves (dashed).

**Figure 7:**
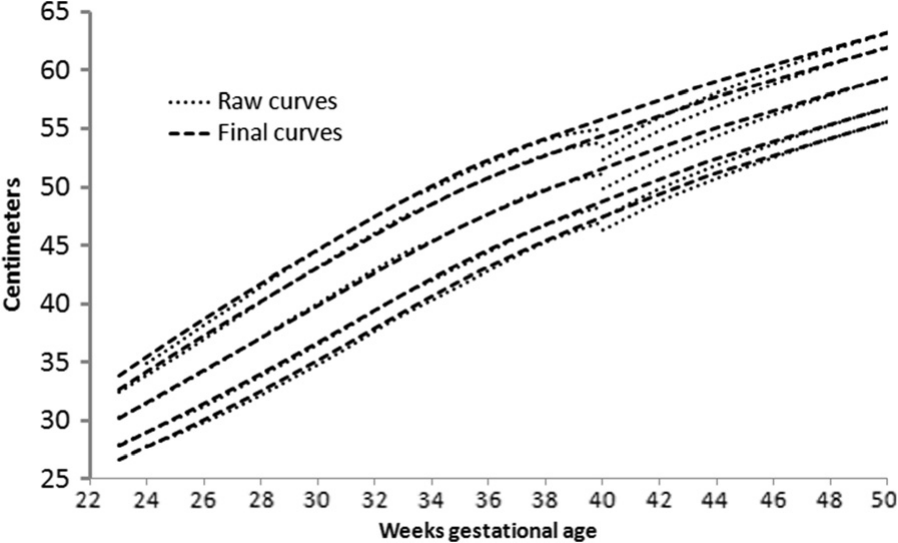
Boys meta-analysis length curves (dotted) with the final smoothed growth chart curves (dashed).

The individual datasets from the literature showed good agreement with each other, especially along the 50th and lower centiles (Figures 1, 2, and 3) and the meta-analysis curves had a close fit with the individual datasets up to 36 weeks and at 50 weeks (Figures 5, 6, 7). The final splined weight curves were within 3% of the meta-analysis curves for 24 through 36 weeks for both genders, except for a 3.8% difference for girls at 32 weeks along the 90th centile. None of the length measurements differed by more than 1.8% percent between the meta-analysis and the splined curves; all weeks of the head circumference curves were within 1.5%. The meta-analyses for head circumference and length for girls and boys were close enough to normal distributions that normal distributions were used to summarize the data. The measures at 50 weeks were within 0.5% of the WHOGS values.

**Figure 8:**
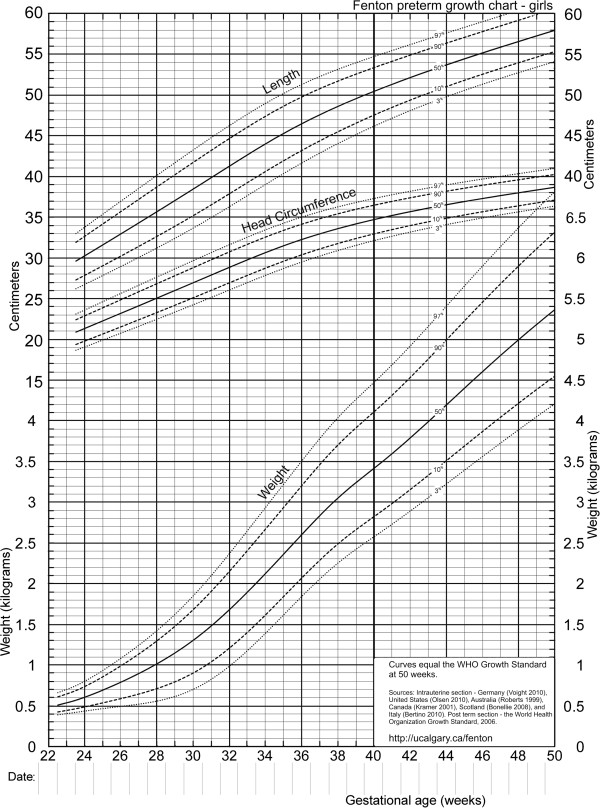
Revised growth chart for girls.

**Figure 9:**
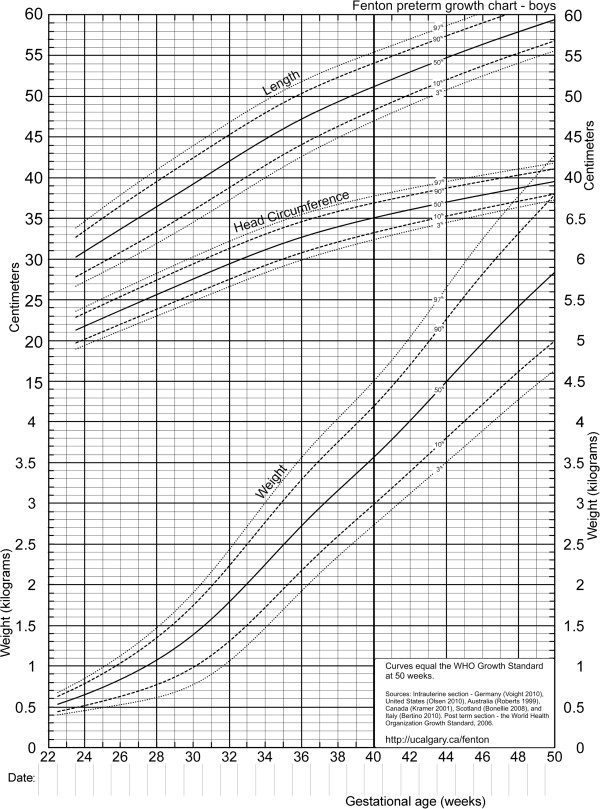
Revised growth chart for boys.

Girl and boy charts were prepared (Figure 8 and 9), by shifting the age by 0.5 weeks to allow plotting by exact age instead of completed weeks. The LMS Parameters [15] were used to develop the exact z-score and percentile calculators for the new growth chart.

**Figure 10:**
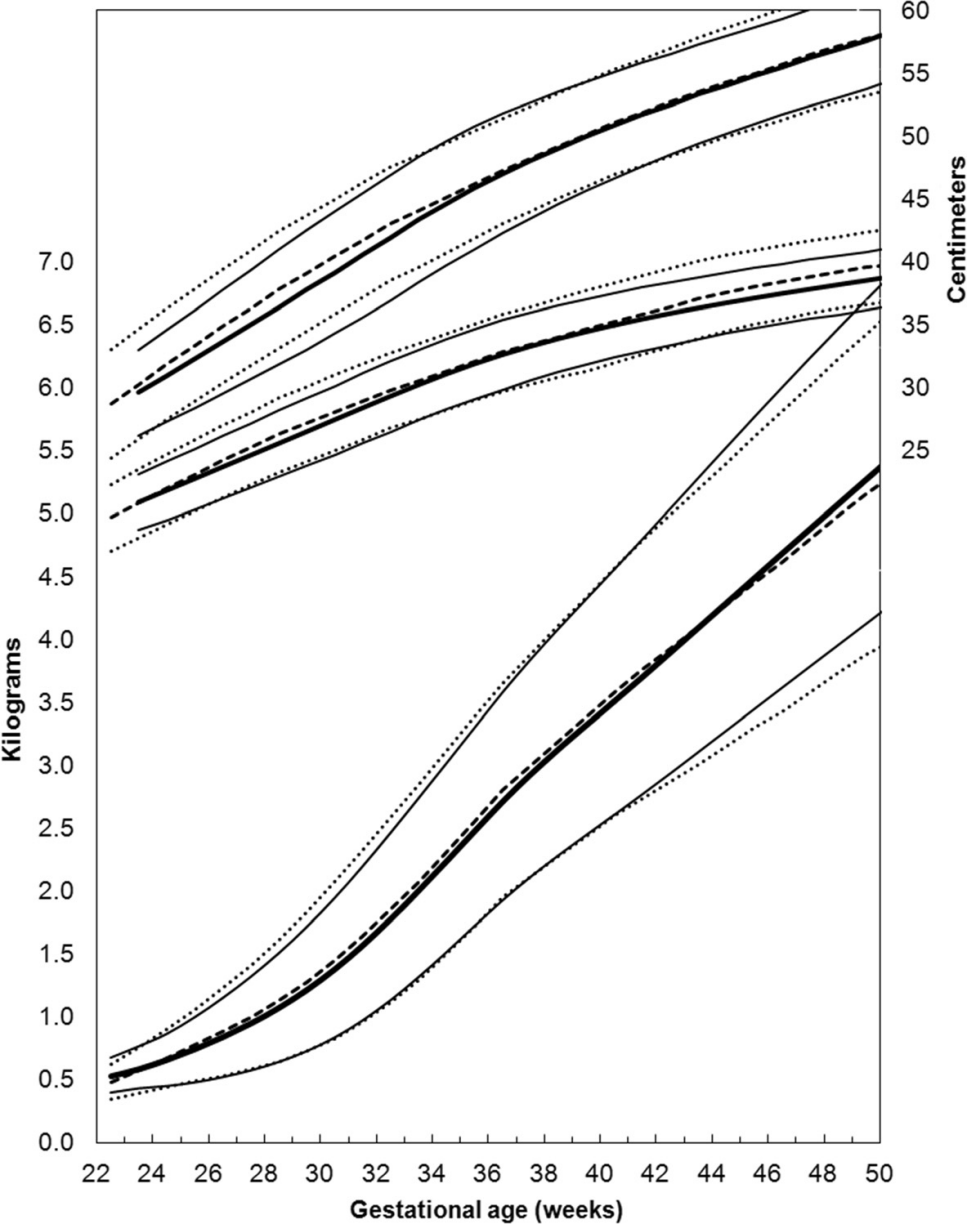
**Comparison of the revised growth chart for girls (solid curves) and the 2003 Fenton growth chart (dashed curves) 3rd, 50th, and 97th percentile curves for length, head circumference, and weight).** Both the 2003 and the revised growth curves are shown shifted to actual weeks.

**Figure 11:**
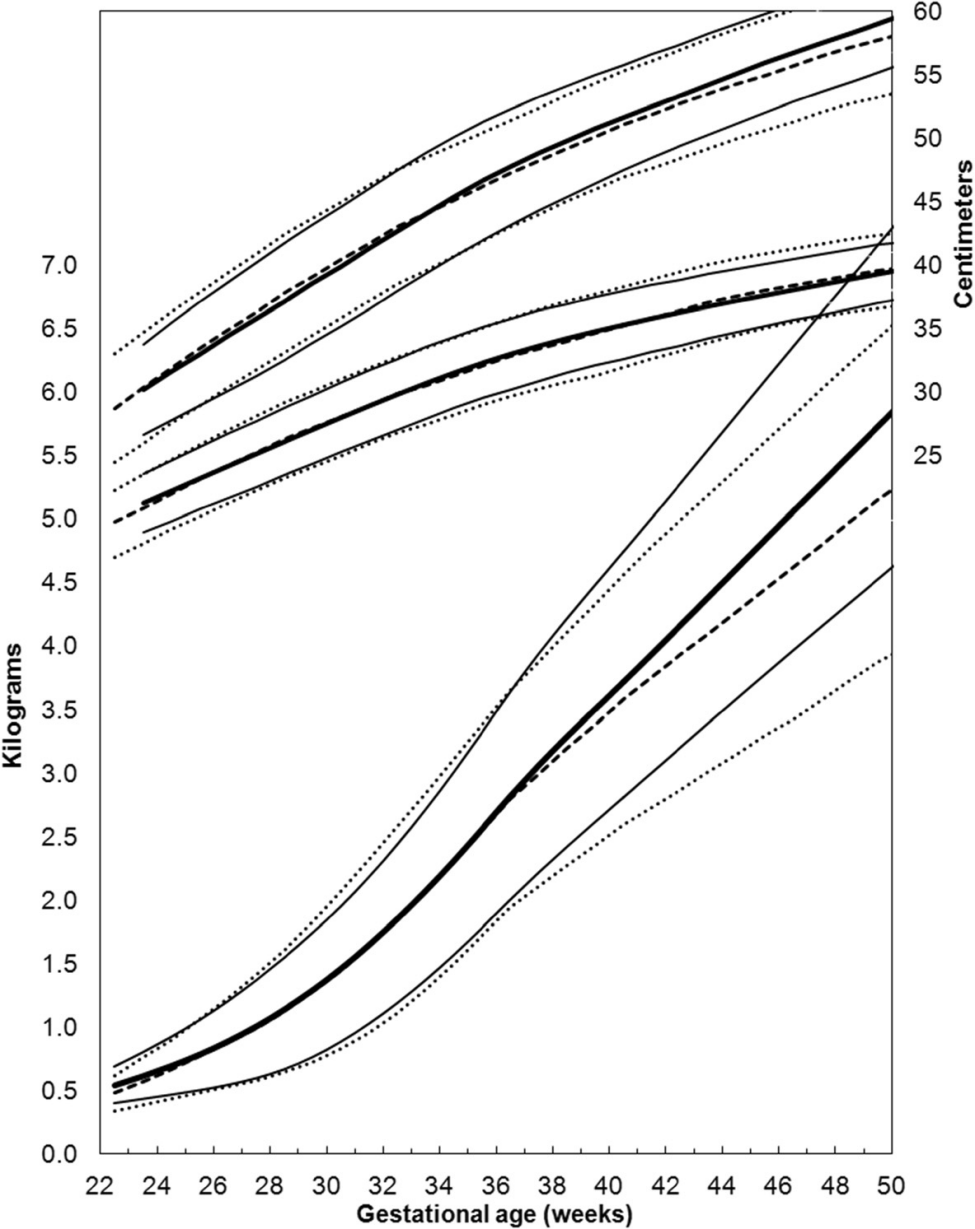
**Comparison of the revised growth chart for boys (solid curves) and the 2003 Fenton growth chart (dashed curves) 3rd, 50th, and 97th percentile curves for length, head circumference, and weight).** Both the 2003 and the revised growth curves are shown shifted to actual weeks.

In the two graphical comparisons between the revised growth charts, one for each sex, with the 2003 Fenton preterm growth chart revealed that the curves were quite similar (Figures 10 and 11). Generally the new girls’ curves were slightly lower (Figure 10) and the new boys’ slightly higher (Figure 11) for all 3 parameters (weight, head circumference, and length) than the 2003 curves. The most dramatic visual and numerical difference between the new charts and the 2003 chart was the higher shift of the boys’ weight curves after 40 weeks compared to the 2003 chart, reaching a maximum difference at 50 weeks of 650, 580, and 740 grams at the 3rd, 50th, and 97th percentiles, respectively. The second biggest visual difference was the lower pattern of the girls’ length curves below 37 weeks; the difference in length reached a maximum numerical value of 1.7 centimeters at 24 weeks along the 97th percentile.

## Discussion

We used a strict set of inclusion criteria to include only the best data available to convert fetal and infant size data into fetal-infant growth charts for preterm infants. The revised sex-specific actual-age (versus completed weeks) growth charts (Figure 9 and 10), are based on birth size information of almost four million births with confirmed or corrected gestational ages, born in developed countries (See Features of the new growth chart). The revised charts are based on the recommended growth goal for preterm infants, the fetus and the term infant, with smoothing of the disjunction between these datasets, based on the findings of our international multicentre validation study (Fenton TR, Nasser R, Eliasziw M, Kim JH, Bilan D, Sauve R: Validating the weight gain of preterm infants between the reference growth curve of the fetus and the term infant, The Preterm Infant Multicentre Growth Study. Submitted BMC Ped 2012). These charts are consistent with the meta-analysis data up to and including 36 weeks, thus they can be used for the assessment of size for gestational age for preterm infants under 37 weeks of gestational age. This growth chart is likely applicable to preterm infants in both developed and developing countries since the data was selected from developed countries to minimize the influence from circumstances that may not have been ideal to support growth.

### Features of the new growth chart

•Based on the recommended growth goal for preterm infants: The fetus and the term infant

•Girl and boy specific charts

•Equivalent to the WHO growth charts at 50 weeks gestational age (10 weeks post term age).

•Large preterm birth sample size of 4 million infants;

•Recent population based surveys collected between 1991 to 2007

•Data from developed countries including Germany, Italy, United States, Australia, Scotland, and Canada

•Curves are consistent with the data to 36 weeks, thus can be used to assign size for gestational age up to and including 36 weeks.

•Chart is designed to enable plotting as infants are measured, not as completed weeks. The x axis was adjusted for this chart so that infant size data can be plotted without age adjustment, i.e. Babies should be plotted as exact ages, that is a baby at 25 3/7 weeks should be plotted along the x axis between 25 and 26 weeks.

•Exact z-score and percentile calculator available for download from http://ucalgary.ca/fenton. Data is available for research upon request.

It may be more intuitive to plot on growth charts using exact ages rather than on the basis of complete weeks. Several years ago, the WHO used completed age for growth chart development [12]. This recommendation was likely due to the way data had been collected in the past, that is all 26 0/7 through 26 6/7 week infants were included in the 26 week completed week category. However, with the use of computers to plot on growth charts comes the potential to more accurately plot measurements to the exact day of data collection. Thus the time scale of the horizontal axes of these new growth charts were re-scaled to actual age, for ease of use and understanding. For example, a baby at 25 3/7 can be intuitively plotted between 25 and 26 weeks.

Exact z-score and centile calculators for the revised charts are available for download: http://ucalgary.ca/fenton. Data is available for research upon request.

The data revealed that between 22 weeks to 50 weeks post menstrual age, the fetus/infant multiplies its weight tenfold, for example, the girls’ median weight increased from a median of 520 to 5360 grams. Using a fetal-infant growth chart allows clinicians to compare preterm infants’ growth to an estimated reference of the fetus and the term infant.

There was a remarkably close fit of the included preterm surveys for weight, head circumference and length from the 6 countries, especially at the 50th percentile, even though the data came from different countries.

The splining procedures we used have produced a chart that has integrity and good agreement with the original data. Smoothing of the LMS parameters is recommended since minor fluctuations are more likely due to sampling errors rather than physiological events [15]. Experts recommend that growth charts be developed based on smoothed L, M and S, to constrain the adjacent curves so that they relate to each other smoothly [15]. The World Health Organization set their L parameter to 1 for head circumference and length, while they maintained the exact L values for infants’ weights [58]. The data under study here revealed the same effect as the WHO data; we found that both head circumference and length were close enough to normal distributions that normal distributions could summarize the data, while the exact L’s were needed to retain the nuances of the weight curves.

The differences between the revised growth charts and the 2003 Fenton preterm growth chart may reflect improvements since the selected preterm growth references for the new versions are more likely globally representative of fetal and infant growth. Some of the differences between the current charts and the 2003 version are likely due to the separation into girl and boy charts, since the shifts of the girls’ curves tend to be downward and the boys’ curves upward. The weight shifts after 40 weeks were upward for both sexes, due to the higher values for the WHOGS compared to the CDC growth reference [59] at 10 weeks post term.

The ideal growth pattern of preterm infants remains undefined. These revised growth charts were developed based on the growth patterns of the fetus (as has been determined by size at birth in the large population studies) and the term infant (based on the WHO Growth Standard) [2]. Ultrasound studies and comparison of subgroups of prematurely born infants suggest that the fetal studies, such as those used in this development, may be biased by the premature birth since fetuses who remain in utero likely differ in important ways from babies who are born early [60,61]. However, fetal size from these imperfect studies may be the best data available at this point in time for comparing the growth of preterm infants since the alternative, to compare to in utero infants requires extrapolation from ultrasound measurements. To use other premature infants as the growth reference for preterm infants may not be ideal since the ideal growth of preterm infants has not been defined, has been changing over time [62], and is influenced by the nutrition and medical care received after birth [63,64].

Although the WHOGS is considered to be a growth standard, the infants in the population-based surveys of size at birth are more likely representative of the reference populations and were not selected to be healthy. Thus these growth charts are growth references and are not a growth standard. The INTERGROWTH study, currently underway, will rectify this problem, since their purpose is to develop prescriptive standards for fetal and preterm growth [65].

## Conclusion

The inclusion of data from a number of developed countries increases the generalizability of the growth chart. The revised preterm growth chart, harmonized with the World Health Organization Growth Standard at 50 weeks, may support an improved transition of preterm infant growth monitoring to the WHO charts.

## Competing interests

The authors declare that they have no competing interests.

## Authors’ contributions

The author’s responsibilities were as follows: JHK suggested the study, TRF & JHK designed the study and conducted independent literature searches, TRF extracted the data, performed the statistical analysis, and wrote the manuscript. Both of the authors contributed to interpret the findings and writing the manuscript, and both authors read and approved the final manuscript.

## Pre-publication history

The pre-publication history for this paper can be accessed here:

http://www.biomedcentral.com/1471-2431/13/59/prepub
